# Role of exosomes in malignant glioma: microRNAs and proteins in pathogenesis and diagnosis

**DOI:** 10.1186/s12964-020-00623-9

**Published:** 2020-08-03

**Authors:** Amir B. Ghaemmaghami, Maryam Mahjoubin-Tehran, Ahmad Movahedpour, Korosh Morshedi, Amirhossein Sheida, Seyed Pouya Taghavi, Hamed Mirzaei, Michael R. Hamblin

**Affiliations:** 1 Department of Psychology, Behaviour, Genetics and Neurobiology Program, University of Toronto, Toronto, Canada; 2 Student Research Committee, Mashhad University of Medical Sciences, Mashhad, Iran; 3 Department of Medical Biotechnology, Faculty of Medicine, Mashhad University of Medical Sciences, Mashhad, Iran; 4 Department of Medical Biotechnology, School of Advanced Medical Sciences and Technologies, Shiraz University of Medical Sciences, Shiraz, Iran; 5 Student research committee, Shiraz University of Medical Sciences, Shiraz, Iran; 6 School of Medicine, Kashan University of Medical Sciences, Kashan, Iran; 7 Research Center for Biochemistry and Nutrition in Metabolic Diseases, Institute for Basic Sciences, Kashan University of Medical Sciences, Kashan, Iran; 8 Wellman Center for Photomedicine, Massachusetts General Hospital, Harvard Medical School, Boston, USA; 9 Laser Research Centre, Faculty of Health Science, University of Johannesburg, Doornfontein, Johannesburg, 2028 South Africa

**Keywords:** Gliomas, Exosomes, MicroRNAs, Proteins, pathogenesis, Therapy, Biomarkers

## Abstract

Malignant gliomas are the most common and deadly type of central nervous system tumors. Despite some advances in treatment, the mean survival time remains only about 1.25 years. Even after surgery, radiotherapy and chemotherapy, gliomas still have a poor prognosis. Exosomes are the most common type of extracellular vesicles with a size range of 30 to 100 nm, and can act as carriers of proteins, RNAs, and other bioactive molecules. Exosomes play a key role in tumorigenesis and resistance to chemotherapy or radiation. Recent evidence has shown that exosomal microRNAs (miRNAs) can be detected in the extracellular microenvironment, and can also be transferred from cell to cell via exosome secretion and uptake. Therefore, many recent studies have focused on exosomal miRNAs as important cellular regulators in various physiological and pathological conditions. A variety of exosomal miRNAs have been implicated in the initiation and progression of gliomas, by activating and/or inhibiting different signaling pathways. Exosomal miRNAs could be used as therapeutic agents to modulate different biological processes in gliomas. Exosomal miRNAs derived from mesenchymal stem cells could also be used for glioma treatment. The present review summarizes the exosomal miRNAs that have been implicated in the pathogenesis, diagnosis and treatment of gliomas. Moreover, exosomal proteins could also be involved in glioma pathogenesis. Exosomal miRNAs and proteins could also serve as non-invasive biomarkers for prognosis and disease monitoring.

Video Abstract

Video Abstract

## Background

Malignant glioma is the most deadly type of brain cancer in humans [1]. It is commonly divided into four grades (I–IV) according to histopathological evaluation. Glioblastoma mutiforme (GBM), grade IV glioma, is the most common and lethal sub-type. Even after the standard treatment methods, including a combination of surgery with radio-chemotherapy [2], the prognosis of GBM IV patients is very poor [3]. According to WHO 2016, conventional histological examination using H&E-stained sections, remains the initial method of stratification, which determines the major categories, such as infiltrating glioma, embryonal tumor or neuronal tumor based on the histology [4]. The median survival for GBM IV patients is only 15 months, and only 3–5% survive more than 36 months [5]. The lack of a non-invasive monitoring procedure to assess the effectiveness of GBM treatment is also a bottleneck for clinical management.

Extracellular vesicles (EVs) are cell membrane-coated vesicles shed from cells that transport cytoplasmic or membrane components to nearby cells and can be detected in body fluids. EVs contain various components such as proteins, lipids, DNAs, mRNAs, and various types of non-coding RNAs [6–8]. Exosomes are a sub-group of EVs with a size range of 30–100 nm. EVs which are released by the direct pathway have often been called “microvesicles”, whereas EVs released by the endocytic pathway have often been termed “exosomes”. They mediate signaling pathways between cancer cells and the other cells in their environment [9, 10].

The transfer of various cargos contained within exosomes is a critical process to mediate cell-cell communications [11]. Exosomes play a vital role in cancer because their contents such as microRNAs (miRNAs), proteins and other physiological compounds vary at different stages of cancer development [12, 13]. miRNAs are non-coding single-stranded RNAs with a length of 18–27 nucleotides. They influence various cellular processes by decreasing the level of translation of their target mRNAs [14]. Recent studies have shown that miRNAs conveyed within exosomes can mediate communication between cancer cells and their milieu [15–17]. Exosomal miRNAs have been associated with glioma pathogenesis via activation and/or inhibition of several signaling pathways. Better understanding of the role of exosomal miRNAs could contribute to the discovery and development of new diagnostic and therapeutic platforms for glioma. The present review summarizes the different exosomal miRNAs and proteins that have been reported to be involved in the pathogenesis of gliomas. We highlight some exosomal miRNAs and proteins that could be used as diagnostic and therapeutic biomarkers in glioma.

## Exosome biogenesis

**Fig. 1:**
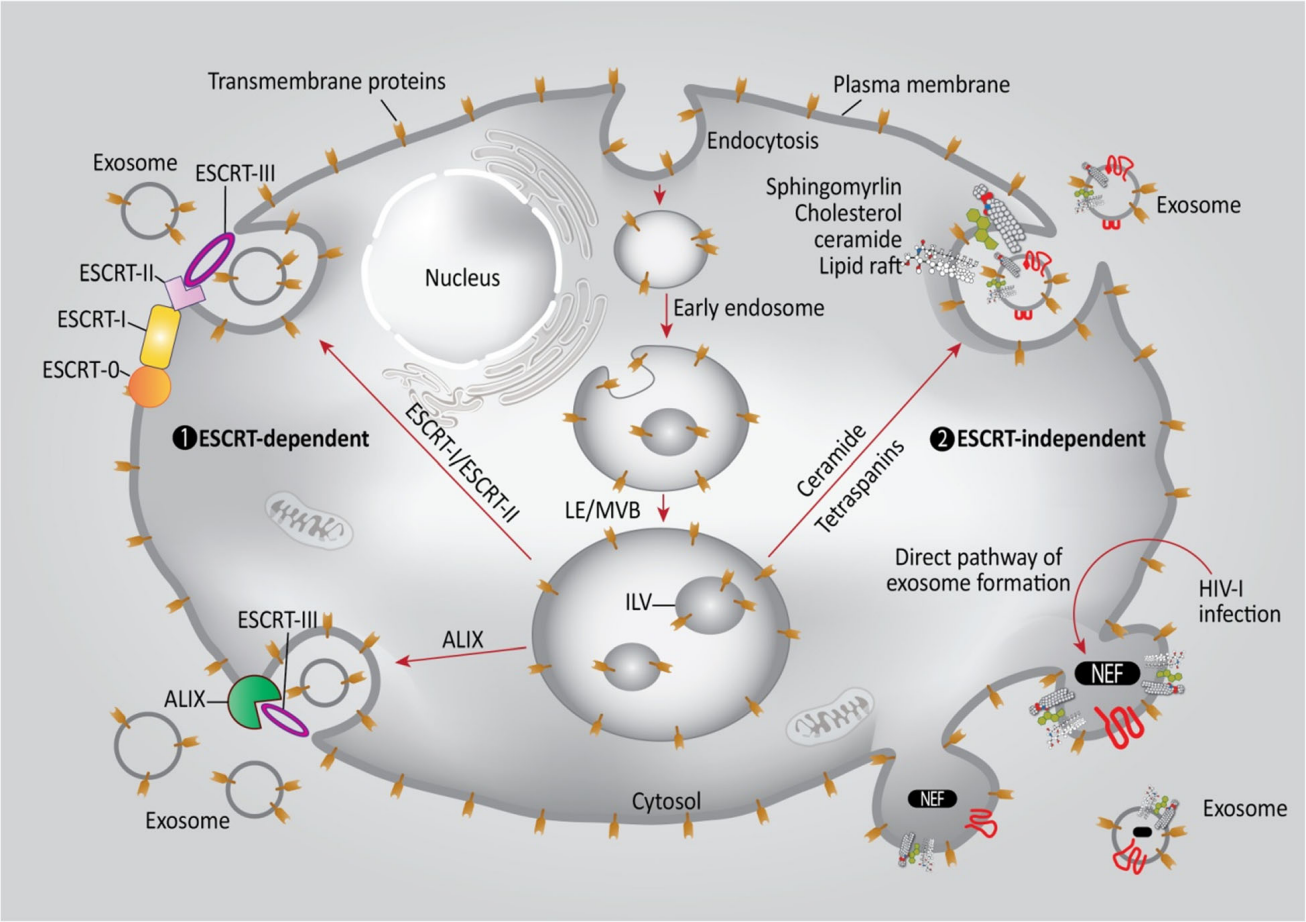
A schematic of exosome formation. Exosome formation is divided into two main pathways: ‘classical’ and ‘direct’. The ‘direct pathway’ involves exosome formation via direct exocytosis of vesicles, such as MVs originating from the external budding of the plasma membrane. The ‘classical pathway’ requires the re-activation of endosomes that originated from the internal budding of the plasma membrane. This pathway results in MVE. Following the active packaging of their components, MVE can fuse with the plasma membrane, and exosomes will then be released to the extracellular space. Exosomes are composed of a lipid bilayer and contain non-coding miRNAs, transmembrane and cytoplasmic proteins, and single-stranded and double-stranded DNA sequences. Exosomes contain proteins such as tetraspanins, ALIX, class-I and -II MHC molecules, and tumor-derived neo-antigens. ALIX: ALG-2 interacting protein X; ESCRT: Endosomal sorting complexes required for transport; LE/MVB: late endosome/multivesicular body; ILV: Intralumenal vesicle; MHC: Major histocompatibility complex; MVE: multi-vesicular endosomes; NEF: Negative Regulatory Factor. Figure adapted from [25]

The criteria for dividing EVs into subtypes are: a) physical characteristics of EVs, such as size or density; b) biochemical composition; or c) descriptions of the conditions or cells of origin [18]. Based on their size and biogenesis, EVs can be divided into three types including: 1) exosomes; 2) microvesicles; and 3) apoptotic bodies [19, 20]. Exosomes are vesicles with a size range 30-100 nm [21]. Exosomes are composed of a common combination of protein and lipid components. The composition is derived from the endosomes from which they originate. The proteins comprise, tetraspanins (CD9, CD63, CD81 and CD82), multivesicular body related proteins (Alix and Tsg101), heat shock proteins (Hsp90 and Hsc70), transport proteins (GTPases, annexins and flotillin), and integrins. The membrane lipids consist of cholesterol, sphingomyelin and ceramide [22–24]. The outer surface of exosomes has many saccharide groups, such as mannose, sialic acid and glycans. The space between the two sides of the lipid membrane is enriched in phosphatidyl ethanolamine [23]. The ESCRT (endosomal sorting complexes required for transport) proteins are involved in exosome generation, as shown in Fig. 1. The ESCRT protein family is divided into four subgroups, i.e., ESCRT-0, ESCRT-I, ESCRT-II, ESCRT-III [26]. These proteins operate in a sequential manner on the cytosolic endosomal surface, which leads to ILV formation via stimulating the inward budding of the membrane, followed by fission​. ESCRT-0 launches the endosomal ESCRT pathway by inducing phosphatidylinositol 3-phosphate to bind to ubiquitin. Hrs and STAM1/2, both of which are ESCRT-0 proteins, play an important role in the binding of the ubiquitinated cargos. The Hrs subunit binds phosphatidylinositol 3-phosphate using its FYVE domain, thereby recruiting ESCRT-0 to pre-MVB endosomes [27]. An ubiquitin-interacting domain is also found in ESCRT-I and ESCRT-II, which sort the ubiquitinated cargos into ILV along with ESCRT-0. The membrane is consequently invaginated and constricted by ESCRT-III which is recruited by ESCRT-I and ESCRT-II [27]. The ubiquitinated cargos of exosomes, which consist of both cytoplasmic and membrane proteins, are organized by ESCRT complexes. Exosomal secretion is highly dependent on TSG101, a subunit of ESCRT-1. MVBs may be triggered by some cargos without interactions with ESCRT-0, −I and –II. An ESCRT-interacting protein, ALIX, binds to the ESCRTIII component CHMP4 and the G protein-coupled membrane receptor PAR1, which, in turn, sorts PAR1 as a cargo to MVBs without requiring ubiquitylation. Additionally, ALIX interacts with the PDZ scaffolding protein syntenin, leading to syndecans binding to CD63 as PDZ ligand cargos. Therefore, ALIX, syntenin, syndecan and CD63 can all be found in MVBs and exosomes without requiring ubiquitination [27, 28]. ESCRT-independent pathways regulate the biogenesis of tetraspanin-containing exosomes and require the participation of lipids. Exosomes are targeted by several proteins via tetraspanins or protein lipidation, involving a glycosylphosphatidyl inositol anchor or saturated fatty acid modifications. Exosomes contains high concentration of tetraspanins with four different transmembrane domains, each possessing a particular palmitoylation site. CD9, CD63, CD37, CD81, CD82 (along with other tetraspanins) are considered to be specific exosome biomarkers, since they are found abundantly on the exosome surface [11]. During the maturation of reticulocytes, exosomes are targeted with three glycosylphosphatidyl inositol-anchored proteins, i.e., CD55, CD58, CD59, and the palmitoylated protein Lyn. Proteins with these lipid modifications enter the lipid rafts consisting of sphingomyelins, cholesterols and ceramides in a selective manner. The lipid rafts accumulate in the exosomal membranes [29, 30]. Cellular exosome release has been reported to be up-regulated by HIV-1 viral infection, which immediately leads to ESCRT-independent biogenesis of exosomes. Nef, a HIV-1 protein anchored to lipid raft micro-domains, has been observed in exosomes from human cells infected with this virus. Two classical exosomal markers, tetraspanins CD63 and CD81 are also found in these exosomes, which have similar sizes to classical exosomes [20, 30]. Exosomes derived from many cell types start off as cargo-containing ILVs within LEs or MVBs. The ESCRT complex, tetraspanins and lipid rafts can all promote exosome biogenesis. Ubiquitinated cargoes are clustered by the ESCRT-0 complex. Membrane budding is mediated by the ESCRT-I and ESCRT-II complexes, and the resulting vesicles are cleaved from the membrane via the “molecular scissors” role of the ESCRT-III complex. High amounts of sphingomyelin, cholesterol, and ceramide are located within the membrane lipid rafts of exosomes. Both endocytic and exocytic processes are associated with the microdomains of highly fluid lipid rafts, and exosome formation is depended on tetraspanin function. Cargoes are selected for further exosome release by tetraspanin family proteins, which possess four transmembrane domains. The ILVs of MVBs and exosomes are enriched with tetraspanins. Moreover, extracellular exosome secretion is promoted by Nef, a HIV-1-encoded protein [25].

## Exosomal microRNAs and gliomas

### Exosomal microRNAs

**Fig. 2:**
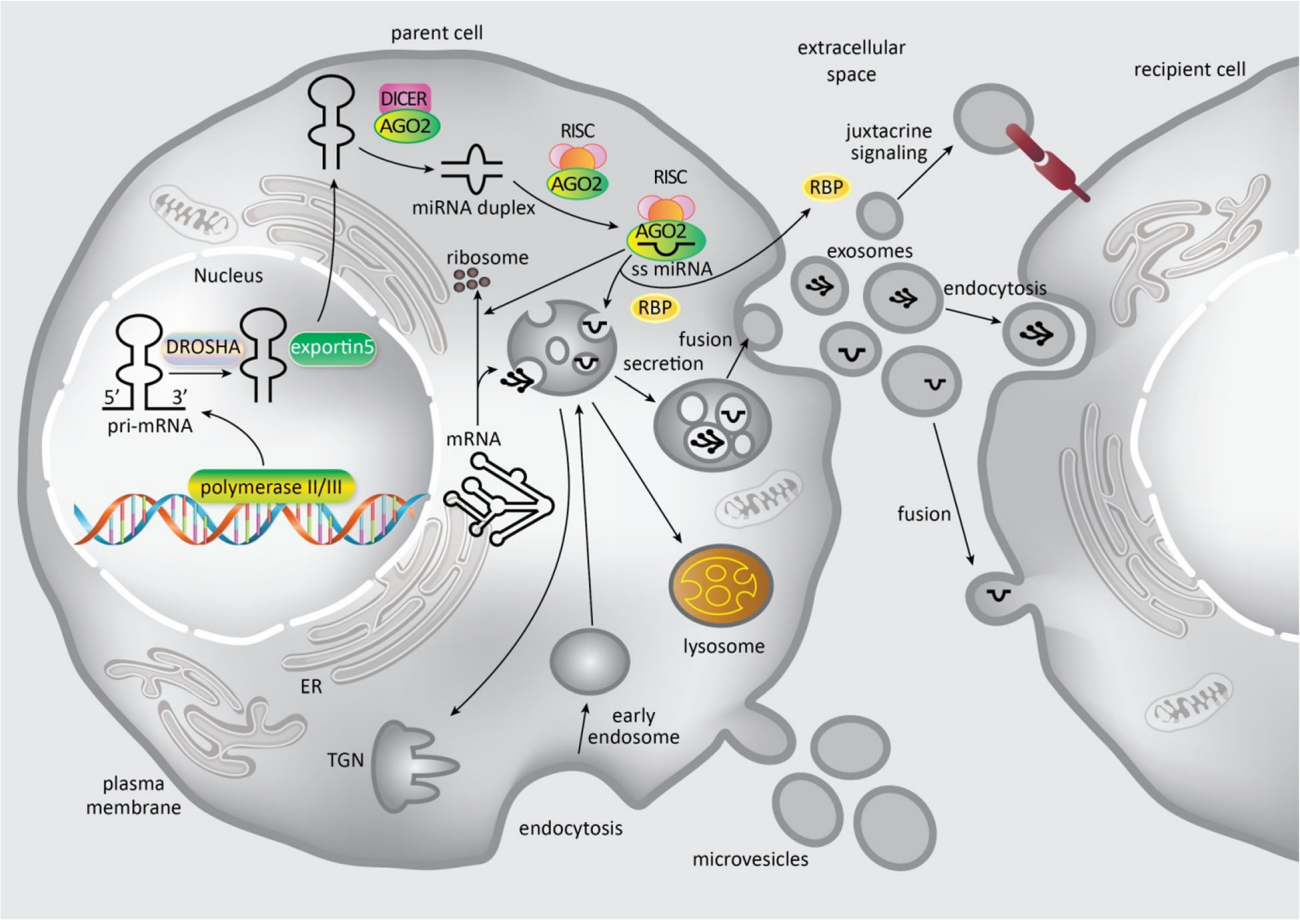
Biogenesis of exosomes within the parent cell and uptake of exosomes by the recipient cell. Vesicular pathways and miRNA/mRNA pathways come together because some RNA molecules are bound within the endosomal limiting membrane in the cytoplasm. RBPs (RNA-binding proteins) translocate miRNA strands into MVBs (multivesicular bodies) for exosome encapsulation, or to the cell membrane for further release. During maturation, the endosomes are transported to the TGN (trans-Golgi network) where they either undergo lysosomal degradation or secrete their intraluminal vesicles (ILVs) via microtubules towards the plasma membrane. Parent cell exosomes may carry out juxtacrine signaling, fusion or endocytosis in order to interact with the recipient cells. Parent cells secrete microvesicles into the extracellular matrix, by outward budding from the cell surface. Figure adapted from [32]

The miRNA genes in the mammalian genome are located within both protein-encoding and non-coding DNA sequences [31]. The RNA polymerase II enzyme transcribes the majority of miRNAs to initially produce long primary miRNAs (pri-miRNAs), and then the RNase III enzymes (Drosha and Dicer) continue the process to create mature miRNAs consisting of 19–24 nucleotide duplexes (Fig. 2) [33]. Dicer transfers the duplex to one of four Argonaute (Ago) proteins. The duplex has a guide strand with its 5′-U head region enriched in A/G nucleotides. This 5′ sequence interacts with Ago for the regulation of the expression of target mRNAs. Furthermore, the duplex has a passenger strand that usually starts with a 5′-C region, which is U/C rich and is designed to be degraded. However, expression profiles in several tissues have suggested that both strands could be equally active [33]. There is an alternative pathway for miRNA processing, which is Drosha-independent. This pathway involves mirtrons (microRNAs located in the introns of the mRNA encoding host genes), and snoRNA- and tRNA-derived miRNAs.

Although some miRNAs can interact with Ago to bind their target mRNA inside the cell, others must be transported to MVBs via RNA-binding proteins (RBPs) and finally loaded into exosomes and secreted from the plasma membrane (Fig. 2 )[17]. Evaluation of the exosomal miRNA profile derived from cardiac fibroblasts showed high amounts of various miRNA passenger strands [34]. The absence of Ago2 within exosomes suggests that exosomal miRNAs are not degraded and/or sorted by other RBPs [35].

### Exosomal microRNAs and glioma resistance to drugs and radiotherapy

Glioblastoma (GBM) has a poor prognosis because it infiltrates and invades into the normal brain parenchyma along vascular tracks. A combination of surgery and radiotherapy and/or chemotherapy is the standard treatment for GBM; unfortunately, cancer recurrence is most commonly observed [36, 37]. Thus, it is necessary to reverse GBM resistance to radiation and/or cytotoxic drugs to find new approaches for treatment. Most solid tumors grow in hypoxic conditions, which plays a vital role in tumor development and resistance to treatment by causing changes in the biology of both cancer and stromal cells [38, 39]. Tumor hypoxia is one of the main contributing factors to the failure of cancer treatment [40], especially in radiotherapy.

Exosomal miRNAs are important in the development of different types of cancer, including gliomas [41]. Yue et al. showed that exosomal miR-301a contributed to glioblastoma resistance to radiotherapy. Hypoxic GBM cells secreted the exosomal miR-301a, which could be transferred to responsive cells that were originally in normoxic conditions, but then became resistant to radiation. Exosomal miR-301a directly targeted TCEAL7 genes, which function as tumor suppressors in GBM progression, and actively suppressed their expression in normoxic glioma cells. TCEAL7 down-regulated the Wnt/β-catenin signaling pathway via inhibiting β-catenin translocation from the cytoplasm to the nucleus. They suggested that the Wnt/β-catenin pathway was triggered by miR-301a-mediated suppression of TCEAL7. This recently discovered exo-miR-301a/TCEAL7-signaling axis could be a new target for reversing tumor cell resistance to radiotherapy in GBM patients [42].

Temozolomide (TMZ) is a DNA-alkylating compound that damages DNA and acts as a cytotoxic drug against GBM. GBM can develop resistance to TMZ thus lessening its effectiveness [43, 44]. TMZ creates DNA double-strand breaks (DSBs) in DNA due to nucleotide damage, which induce apoptosis by caspase-dependent pathways [45, 46]. It has been demonstrated that DSBs are often repaired via non-homologous end-joining (NHEJ) [44, 46, 47]. The XRCC4 protein combines with DNA ligase IV and forms a heterodimeric complex that mediates the NHEJ process. This complex can join together the broken ends of the DNA double strand and help damaged cells survive [43, 44]. It has been reported that the NHEJ pathway has a role in governing the sensitivity of GBM cells to TMZ [44]. It has also been shown that glioma sensitivity to TMZ is related to polymorphisms in the XRCC4 gene [48]. The expression of the XRCC4 gene is considerably down-regulated in many glioma cells, confirming the critical role of XRCC4 in brain tumors [49]. However, more research is still needed on the function of XRCC4 in the oncogenicity and TMZ resistance in GBM.

Zeng et al. evaluated whether exosomal miRNAs could contribute to TMZ-resistance in GBM cells [50]. The expression level of miR-151a was measured using quantitative PCR in two TMZ-resistant GBM cell lines. A RNA chromatin immunoprecipitation (RNA-ChIP) assay, combined with bioinformatics and microarray assays were used to identify the main targets for miR-151a. The exosomes isolated from cell lines, serum and cerebrospinal fluid (CSF) were investigated, and their effects on resistance to TMZ in target GBM cells were evaluated. A reduction in miR-151a resulted in higher resistance to TMZ. Conversely over expression of miR-151a sensitized chemo-resistant GBM tumor cells to TMZ by suppressing the XRCC4-DNA repair pathway. GBM chemo-resistant cells have a lower content of miR-151a containing exosomes, which induces resistance to TMZ. Restoration of the secretion of miR-151a containing exosomes by the resistant cells eliminated resistance to TMZ. Levels of miRNA-151a containing exosomes in CSF reflected the chemo-resistance of the GBM tumor. Therefore sampling of exosomal miR-151a could act as a ‘liquid biopsy’ in a non-invasive manner for evaluation of chemo-resistance. These exosomes could also be a component of a treatment for refractory GBM tumors [50].

Several reports have suggested that the miR-155HG/miR-155 could have an important role in GBM development, and that NSC141562 which acts as a miR-155/miR-155 repressor, could be a part of GBM treatment [51]. Shi et al. showed that miR-1238 could play a tumor suppressor role in non-small cell lung cancer via targeting LHX2 and inhibiting proliferation [52]. Conversely, Yin et al. showed that over-expression of miR-1238 plays an important role in mediating the acquired TMZ resistance in GBM patients [53]. Down-regulation of miR-1238 in TMZ resistant cells could sensitize resistant GBM cells by directly targeting the CAV1/EGFR pathway. Bioactive miR-1238 present in exosomes shed from TMZ-resistant cells could be taken up by TMZ-sensitive cells, thus further spreading TMZ resistance [53]. It has been reported that EGFR has a critical role in resistance to TMZ [54, 55]. A combination of TMZ plus erlotinib (an EGFR kinase inhibitor) could increase survival in GBM patients in comparison with TMZ alone [56]. Using co-immunoprecipitation and confocal microscopy, it was found that EGFR and CAV1 have mutual interactions [53]. The EGFR-PI3K-Akt-mTOR signaling pathway could be activated due to lack of CAV1 binding, resulting in GBM tumor cells developing resistance to TMZ. Therefore exosomal miR-1238 could not only be a prognostic biomarker for assessment of chemotherapy treatment protocols, but could also be an ideal for a novel GBM treatment [53, 57].

### Exosomal microRNAs and other glioma-related processes

Metastasis plays a critical role in most cancer related deaths [58]. GBM is one of the most lethal cancers worldwide, but mainly spreads by local invasion into the brain, rather than by distant metastasis. However GBM metastasis into the human central nervous system can occur, and it makes surgical removal even more difficult [59]. Cells from advanced cancers can secrete exosomes containing onco-proteins, long non-coding RNAs or miRNAs, which all promote tumor development [60, 61]. Many factors such as signaling between various cells, blood vessels, stroma, extracellular matrix and secreted molecules play important roles in the tumorigenesis and development of GBM [62]. There are controversies about the role of exosomes in GBM development. Recently, one study showed that miR-5096 could stimulate the production of filamentous pseudopodia and increase the invasiveness of glioma cells, through the regulation of the K+ channel Kir4.1. miR-5096 could also increase the release of exosomes resulting in GBM metastasis [63].

The Cancer Genome Atlas (TCGA) has suggested that miR-148a may increase the risk of GBM development [64]. Cell adhesion molecule 1 (CADM1) is a neural tissue-specific protein which plays a vital role in cell-cell adhesion between identical and non-identical cells based on the Ca^2+^ concentration [65]. It has been found that CADM1 is a tumor suppressor factor and its expression is suppressed in GBM tumor cells. The CADM1 promoter was hypermethylated in the T98G GBM cell line [66]. CADM1 could suppress activation of STAT3 signaling through interaction with HER2 and Itgα6β4 [67]. STAT3 signaling is commonly activated in GBM cells, and suppression of STAT3 phosphorylation could significantly reduce metastasis [68, 69]. Since the STAT3 pathway could stimulate metastasis and progression in GBM cells, miR-148a could accelerate the progression of GBM by increasing CADM1/STAT3 signaling [70, 71]. Cai et al.*,* found that the levels of miR-148a contained in exosomes in body fluids of GBM patients was higher than healthy individuals [72]. In the T98G cell line, suppression of miR-148a expression resulted in inhibition of cancer development and metastasis. Furthermore, they found that CADM1 could be a target for miR-148a, according to results from a luciferase reporter assay. A reduction was shown for mRNA and protein amounts of CADM1 in GBM tumor tissues. Down-regulation of CADM1 expression in GBM patient samples was closely related to exosomal miR-148a. Furthermore, a miR-148a antagonist activated STAT3 signaling through an increase in the STAT3 protein concentration. Finally, they found that miR-148a containing exosomes could stimulate tumor development and metastasis by activation of STAT3 signaling via CADM1. They proposed that exosomal miR-148a could be a prognostic factor or a target for GBM treatment [72].

Myeloid-derived suppressor cells (MDSCs) are a diverse population of naive myeloid cells that are characterized by the CD11b + Gr-1+ phenotype in mice, and the CD14 + HLA-DRlow/−phenotype in humans. MDSCs are produced in the bone marrow and are derived from myeloid progenitor cells, and functional MDSCs carry out robust inhibition of T cell function. Their immunosuppressive function is linked to their ability to generate high amounts of arginase-1, nitric oxide (NO), reactive oxygen species (ROS) and to release IL-10 and transforming growth factor β (TGF-β) [73]. The differentiation and function of MDSCs is governed by activation signals, because the immunosuppressive type of MDSCs is found in cancerous mice but not in healthy mice [73, 74]. Guo et al., identified that glioma cells in a hypoxic condition can secrete miR-29a and miR-92a containing exosomes, which induce the differentiation of functional MDSCs [75]. They reported that glioma-derived exosomes (GEXs) could increase active MDSC differentiation both in vitro and in vivo. Furthermore, hypoxia-induced GEXs (H-GEXs) induced MDSCs more strongly than normoxia-induced GEXs (N-GEXs). A miRNA sequencing study of N-GEXs and H-GEXs, showed that miR-29a and miR-92a containing exosomes which were secreted under hypoxic conditions could induce the proliferation of MDSCs. miR-29a and miR-92a induced the propagation and activation of MDSCs by a direct effect on high-mobility group box transcription factor 1 (Hbp1) and the protein kinase cAMP-dependent type I regulatory subunit alpha (Prkar1a). It was found that gliomas secreted miRNA containing exosomes which induced an immunosuppressive condition in the tumor microenvironment, and that miR-29a/miR-92a containing exosomes could exert regulatory effects on the function of MDSCs [75].

miR-21 is a well-known miRNA that is up-regulated in nearly all cancer types, and stimulates tumor cell proliferation, invasion and metastasis. PDCD4, TIMP3, and RECK are important regulators for apoptosis and metastasis, are also targets for miR-21 [76–82]. Because miR-21 is well-known for stimulating tumorigenesis, it has been considered to be an interesting target for GBM treatment. Suppression of miR-21 by various approaches has been shown to increase apoptosis, radio−/chemo-sensitivity, and to reduce tumor proliferation [83–87]. It was found that miRNA suppression (via either a decoy or a sponge molecule) could be useful for cancer treatment. The sponge-shaped molecule could interact with miRNA(s) or their originating sequences, and could hinder the binding of the miRNA to mRNA [88–90].

Monfared et al., studied whether down-regulation of miR-21 could affect U87-MG and C6 glioma tumor cell lines. They engineered exosomes by loading them with a molecule that sponged miR-21, and then added them to the cells [91]. Their results showed that the engineered exosomes could down-regulate miR-21, and consequently PDCD4 and RECK which are the miR-21 targets were over-expressed. Cells that were treated by sponge-loaded exosomes showed a decrease in proliferation and also increased apoptosis. Lastly, the miR-21-sponge construct loaded into exosomes induced a significant decrease in tumor volume in a rat model of GBM. Taken together, the results showed that administration of engineered exosomes containing miR-21-sponge constructs could be a novel treatment for GMB [91].

### Exosomal microRNAs derived from mesenchymal stem cells in glioma

Researchers have found that mesenchymal stem cells (MSCs) play a vital role in tumor progression and development [92]. Furthermore, bone marrow-derived adult human mesenchymal stem cells (hMSCs) can differentiate into various mesenchymal cell types [93]. Additionally, some studies have suggested that MSCs could be beneficial for GBM treatment [94]. Moreover, stem cells have a high capacity to secrete exosomes. The released exosomes can act as biomarkers of the paracrine secretion of diverse factors produced by MSCs [95]. miR-133b has been shown to play a tumor suppressor role in many cancers [96]. Furthermore, miR-133b also acts as an inhibitor for GBM [97]. Li et al., found that miR-133b was involved in glioma growth and metastasis by its regulatory effect on Sirt1 gene expression [98]. It was shown that EZH2 is present in many different organisms and is over-expressed in various types of cancer [99]. Abnormal up-regulation of the EZH2 gene in glioma cells induces invasion and metastasis in GBM [100]. Furthermore, down-regulation of EZH2 suppresses glioma growth through a negative regulatory effect on the β-catenin signaling pathway [101]. The Wnt/β-catenin pathway is involved in the growth of the central nervous system, and acts as a tumor-promoting pathway in some cancers [102] and is also involved in GBM development [103]. Xu et al., studied the effects of MSC-derived exosomal miR-133b on glioma cell behavior [104]. Microarray assays revealed differentially expressed genes within glioma cells. miR-133b was down-regulated and EZH2 was simultaneously up-regulated, leading to the conclusion that EZH2 is down-regulated by miR-133b. MSC-derived exosomal miR-133b suppressed EZH2, and inhibited the development, invasion, and metastasis of GBM by affecting the Wnt/β-catenin pathway. Additionally, in vivo studies verified the ability of MSC-derived exosomes loaded with miR-133b to inhibit glioma tumor growth. Finally, MSC-derived exosomal miR-133b and the Wnt/β-catenin/EZH2 pathway could act as biomarkers for monitoring and prognosis in glioma therapy [104].

The ADP-ribosylation factor GTPase-activating protein (Arf GAP) catalyzes the hydrolysis of GTP by binding to Arf (a GTP-binding protein of the Ras superfamily), which is followed by alterations in various cellular functions [105]. Arf GAPs have a critical role in membrane vesicle formation through facilitating the transportation of molecules between different cellular organelles [106]. Arf GTPase-activating protein-2 (AGAP2) mediates endosome trafficking and has been shown to be up-regulated in various cancers [107]. Yu et al., showed that MSC-derived exosomal miR-199a could suppress glioma proliferation via decreasing the expression of ArfGAP (which possesses a GTPase domain), an ankyrin repeat and a PH domain 2 (AGAP2) [108]. The expression levels of miR-199a and AGAP2 in glioma cells were evaluated using qPCR, immunohistochemistry and Western blotting. A miR-199a mimic transfected into MSCs, and their secreted exosomes were added to U251 cells. The biological function and chemo-sensitivity of U251 cells were evaluated to study the role of miR-199a/AGAP2 in glioma. miR-199a was down-regulated and AGAP2 was up-regulated in glioma cells. MSC-derived exosomal miR-199a suppressed development, invasion and metastasis in recipient glioma cells. Moreover, addition of MSC-exosomal miR-199a increased glioma cell sensitivity to TMZ, and inhibited tumor growth in vivo, by exerting a negative regulative effect on AGAP2 expression [108].

miR-584 is another tumor suppressor which can inhibit cancer cell proliferation, invasion and migration. It was found that miRNA-584 down-regulates several oncogenes. Furthermore, miRNA-584 can induce apoptosis via inhibiting gene expression of anti-apoptotic proteins [109–112]. miR-584 affects the expression of CYP2J2, which is related to the development of metastasis. A study by Kim et al. specifically analyzed the role of miR-584 in the progression of glioma [113]. They studied this phenomenon by adding miRNA-containing exosomes to the media of cultured MSCs, which had been transfected by a miRNA mimic. This study evaluated the proliferation and invasion of tumor cells in vitro, and quantified the expression of proteins that related to apoptosis, growth, and metastasis. They carried out in vivo experiments, in which U87 tumor cells which had been exposed to miRNA-584-5p transfected MSC derived exosomes were inoculated into mice. The aim of this study was to confirm the ability of miRNA containing exosomes to inhibit the progression of glioma tumors. The results suggested that miRNA transfected MSCs-derived exosomes could be a novel treatment approach for glioma [113].

### Exosomal microRNAs as biomarkers in glioma

The assessment and monitoring of the response to glioma treatment in the neuro-oncology field remains challenging [114]. Radiography and neuro-imaging methods are not sensitive enough to detect the early stages of tumor recurrence or progression. Furthermore, distinguishing pseudo-progression and pseudo-responses from their real counterparts is challenging. Histological analysis of brain biopsy samples can absolutely diagnose and evaluate tumor development, but numerous and repeated brain biopsies are not desirable due to surgical considerations. Moreover, biopsy specimens may not fully represent all the GBM cells that are present with their genetic diversity. Improved non-invasive evaluation of tumors would be a critical step to improve care for GBM patients. More reliable biomarkers are required to monitor treatment progress in a safe, accurate, and time-saving manner before clinical symptoms become apparent. The term “liquid biopsy” refers to a novel approach to assess the GBM tumor burden, and monitor the response to therapy. If this type of test could be developed in the future, it could be an alternative to common diagnostic procedures.

Exosomes are starting to be investigated as sources of biomarkers that can be non-invasively obtained, and can be used for the diagnosis and follow up of many diseases, including cancer [115]. GBM-secreted exosomes are wide-spread in some body fluids and could be sources for identifying nucleic acids and other cancer-related biomarkers [116]. The protein and nucleic acid expression profiles of GBM-derived exosomes have been investigated [117, 118]. Both the exosomal miRNAs (Table 1) and proteins (Table 2) could have important role in glioma. Studies indicated that GBM-derived exosomes contained many small non-coding RNAs (sncRNAs) [168]. sncRNA sequencing showed that some novel sncRNAs were present in GBM-derived exosomes [168]. Studies have shown that some miRNAs, such as miR-9 [119], miR-10a and miR-2 1[120], miR-222 and miR-124-3p [121], miR-125b [122], mir-2 1[123], miR-124a [124], miR-451 and miR-2 1[125], miR-22 1[126], miR-103 and miR-125 [127], miR-302-367 [128], miR-1290 and miR-1246 [129] were up-regulated, but other miRNAs, such as miR-1587 [130], miR-375 [131], miR-454-3p [132], miR-1246 [134], miR-146b [133], and miR-124 [135] were down-regulated in glioma tumors (Table 1). Although the miRNA content of GBM-derived exosomes is related to the source cell, there is likely to be a unique exosomal miRNA profile.

**Table 1:** Role of exosomal miRNAs in gliomas

Exosomal microRNA	Expression status	Target	Notes	Ref
miR-301a	Up	TCEAL7	TCEAL7 is a tumor suppressor in GBM. TCEAL7 is able to modulate the Wnt/β-catenin pathway by blocking β-catenin translocation from the cytoplasm to the nucleus.	[42]
miR-151a	Up	XRCC4	miR-151a reduces XRCC4 levels, induces delay of DSB clearance and encourages cells to become sensitive to TMZ.	[50]
miR-1238	Up	CAV1	The loss of miR-1238 can sensitize resistant GBM cells by directly targeting the CAV1/EGFR pathway.	[53]
mir-5096	Down	Kir4.1, AQP-4	miR-5096 was down-regulated in gliomas.	[63]
miR-148a	Down	CADM1	miR-148a promotes proliferation and metastasis via targeting CADM1 to activate the STAT3 pathway.	[72]
miR-29a	Up	Hbp1	miR-29a was up-regulated in gliomas.	[75]
miR-92a	Up	Prkar1a	miR-92a was up-regulated in gliomas.	[75]
miR-133b	Down	EZH2	miR-133b represses proliferation, invasion, and migration by inhibiting EZH2 and the Wnt/β-catenin signaling pathway.	[104]
miR-199a	Down	AGAP2	miR-199a inhibits glioma progression by down-regulating AGAP2	[108]
miRNA-584-5p	Up	CYP2J2	miRNA-584-5p reduces proliferation and invasion of glioma cells.	[113]
miR-9	Up	COL18A1, THBS2, PTCH1 and PHD3	miR-9 increases angiogenesis.	[119]
miR-10a and miR-21	Up	RORA, PTEN	The hypoxia-inducible expression of miR-10a and miR-21 in GDEs mediates MDSC expansion and activation by targeting RAR-related orphan receptor alpha (RORA) and phosphatase and tensin homolog (PTEN).	[120]
miR-21, miR-222 and miR-124-3p	Up	–	miR-21, miR-222 and miR-124-3p were up-regulated in gliomas.	[121]
miR-125b	Up	–	miR-125b was up-regulated in gliomas	[122]
mir-21	Up	VEGF	mir-21 was up-regulated in gliomas	[123]
miR-124a	Up	FOXA2	miR-124a acts by silencing FOXA2, resulting in aberrant intracellular lipid accumulation	[124]
miR-451, miR-21	Up	c-Myc	–	[125]
miR-221	Up	–	miR-221 was up-regulated in gliomas.	[126]
miR-21, miR-103, miR-24, and miR-125	Up	–	miR-21, miR-103, miR-24, and miR-125 were up-regulated in gliomas.	[127]
miR-302-367	Up	CXCR4/SDF1, SHH, cyclin D, cyclin A and E2F1	Large amounts of miR-302-367 were found in exosomes, which were internalized by neighboring GBM cells.	[128]
miR-1290, miR-1246	Up	–	miR-1290 and miR-1246 were up-regulated in gliomas.	[129]
miR-1587	Down	NCOR1	miR-1587 down-regulates the tumor-suppressive nuclear receptor co-repressor NCOR1	[130]
miR-375	Down	SLC31A1	miR-375 increases apoptosis while suppressing proliferation, migration and invasion. Inhibits glioma cell progression through SLC31A1 suppression	[131]
miR-454-3p	Down	ATG12	miR-454-3p suppresses cell proliferation, migration, invasion, and autophagy in glioma.	[132]
miR-146b	Down	EGFR and NF-κB	miR-146b decreases EGFR and NF-κB protein in 9 L glioma cells in vitro	[133]
miR-1246	Down	TERF2IP	miR-1246 activates the STAT3 signaling pathway and inhibits the NF-κB signaling pathway.	[134]
miR-124	Down	CDK6	miR-124 decreases the migration of GBM cells	[135]
miR-328-3p, miR-339-5p, miR-340-5p, miR-485-3p,, and miR-543	Up	–	miR-328-3p, miR-339-5p, miR-340-5p, miR-485-3p,, and miR-543 were up-regulated in gliomas.	[136]
miR-182-5p, miR-486-5p	Down	–	miR-182-5p and miR-486-5p were down-regulated in gliomas.	[136]
miR-301a	Up	PTEN	miR-301a up-regulates PTEN.	[137]
miR-221	Up	DNM3	miR-221 up-regulates DNM3.	[138]
miR-26a	Up	PTEN	miR-26a increases proliferation and angiogenesis.	[139]

**Table 2:** Role of exosomal proteins in glioma

Exosomal protein	Expression status	Target	Note	Ref
HMGB1	Up	SASH1	HMGB1 plays different roles depending on its location: as an extracellular protein, HMGB1 decreases SASH1 expression, but as an exosomal protein, HMGB1 increases SASH1 expression	[140]
IL-8, PDGFs, caveolin 1, and lysyl oxidase	Up	**–**	The exosomal pathway constitutes a potential target that drives hypoxia-dependent intercellular signaling during tumor development.	[141]
L1CAM	Up	FGFR, FAK	Increases cell motility, proliferation, and invasiveness.	[142]
STC1, STC2	Up	**–**	Induces cell migration in a hypoxia-dependent manner	[143]
EGFRvIII	Up	CD44, BSG, CD151, CD81 and CD82	–	[144]
VEGF-A	Up	claudin-5 and occluding	Increases the permeability of the BBB in vitro by interrupting the expression of claudin-5 and occludin. In vivo permeability assay showed hypoxic GBM-derived exosomes remained functional in the blood circulation and induced permeability in the BBB.	[145]
CRYAB	Up	–	The U373 glioma cells produce and secrete cryAB in exosomes; stimulation with IL-1β and TNF-α significantly increased the levels of cryAB not only in cells but also in secreted exosomes.	[146]
PTRF	Up	Cavin1	PTRF over-expression increases exosome secretion and induces cell growth in vitro. Clinical samples showed a positive correlation between tumor grade and PTRF expression in both tumor tissue and exosomes isolated from blood harvested from glioma patients.	[147]
PD-1	Up	–	–	[148]
*IL-8, ZAP70, TGF-*β	down	ELISPOT, IL-13R,	–	[148]
IL13Rα2, IL13QD	Up	–	Specific binding of IL13QD to tumor associated exosomes was confirmed.	[149]
**CAV1**	Up	p-ERK1/2	Exosome uptake appears to be dependent on intact ERK1/2-HSP27 signaling, and ERK1/2 phosphorylation was negatively influenced by CAV1 during internalization of exosomes.	[150]
NK-Exo	Up	CD63, Alix	In vivo NK-Exo treatment inhibited tumor xenograft growth compared to control mice, and pretreatment of mice with dextran sulfate 2 h before NK-Exo treatment increased the antitumor effect of NK-Exo compared to control and NK-Exo-alone-treated mice.	[151]
SRSF1, SRSF3	Up	PTBP1,PTBP2	–	[152]
NANOGP8	Up	–	–	[153]
IFN-gamma, granzyme B	Down	–	Granzyme B was significantly inhibited in CD8 + T cells exposed to GL26 cell-derived exosomes, and the exosomes could not inhibit the expression of granzyme B in CD4 + T cells and NK cells.	[154]
PTENP1	Up	miR-10a-5p	The lncRNA PTENP1 could be packaged into exosomes from hUC-MSCs, transferred to U87 cells, and then stabilized PTEN by competitively binding miR-10a-5p.	[155]
CLIC1	Up	GFP, FLAG-tagged	CLIC1 is a circulating protein, secreted via extracellular vehicles (Evs) released by either cell lines or GBM-derived CSCs.	[156]
K-Ras	Up	Raf-RBD	–	[157]
immunoglobulin (Ig) G2 and IgG4	Up	CD163	–	[158]
TrkB	Up	YKL-40	Inhibits tumor growth in vivo. Plays a key role in the control of GBM progression and aggressiveness.	[159]
MGMT mRNA	Up	–	–	[160]
EGFRvIII	Up	CD81	EGFRvIII expression either in exosomes or tissue was correlated with poor survival.	[161]
N-glycoproteins	Up	Glycopeptide	329 N-glycosylation sites corresponding to 180 different N-glycoproteins were enriched and identified in plasma exosomes of glioma patients and healthy subjects.	[162]
LOX, ADAMTS1, TSP1, VEGF	Up	KCNJ3	Induces differential gene expression in recipient glioma cells	[163]
CRCL	Down	T cell	Anti-tumor activity through modulating Cbl-b and c-Cbl signaling.	[164]
NF-κB	Up	green fluorescent protein	NF-κB inducible promoter mediates widespread reporter gene expression in tumor-associated myeloid-derived cells after systemic injection of exo-AAV in brain tumor-bearing mice	[165]
Glut-1, HK-2, and PKM-2	Up	MMP-2, MMP-9	Increases glucose consumption and generation of lactate and ATP.	[166]
TDP-43	Up	–	–	[167]

In one study, miRNA-containing exosomes were isolated from the sera of GBM (*n* = 12) patients, and their nucleic acid contents were sequenced [136]. Results from the specimens from grade II-III (*n* = 10) glioma patients were compared to healthy controls, which were age- and gender-matched to patients. Significant differences were found in miRNA expression levels, and the predictive power of individual miRNAs and subsets of miRNAs was assayed by univariate and multivariate statistical analyses. Further analysis based on specimens from GBM patients (*n* = 4) and independent sets of samples from healthy individuals (*n* = 9) and non-glioma cancer patients (n = 10) as controls, were analyzed to measure the specificity and predictive power of this miRNA-based diagnostic assay. In total, 26 different miRNAs were detected in exosomes extracted from sera obtained from GBM patients compared to healthy controls. Seven miRNAs (miR-182-5p, miR-328-3p, miR-339-5p, miR-340-5p, miR-485-3p, miR-486-5p, and miR-543) were chosen as the most stable candidates for detecting GBM, according to random forest modeling and data partitioning. Based on the aforementioned studies, the measurement of six individual miRNA sequences could discriminate GBM patients from healthy controls with the required precision. Seven miRNA biomarkers were able to properly identify all the positive specimens in validation cohorts (*n* = 23). Moreover, 23 dysregulated miRNAs were found in samples of IDH^MUT,^ a lower-grade glioma. miRNAs detected in serum could be used to diagnose GBM with greater precision. It was found that exosomal miRNA profiles are different from formerly described “free-circulating” miRNAs in GBM patients, and appear to be superior for diagnostic purposes [136].

Lan et al., reported that exosomes containing miR-301a, which were isolated from patients with grade IV GBM, were biologically active. They showed that the proliferation and invasion of H4 glioma cells were increased by addition of miR-301a exosomes [137]. The study also found that exosomal miR-301a was over-expressed in the sera of glioma patients in comparison with healthy individuals. The elevated levels of miR-301a containing exosomes were related to increased tumor grade and lower Karnofsky performance status scores. The levels of miR-301a containing exosomes in the serum were significantly decreased after surgery of the primary tumor, and were elevated again after GBM relapse. Kaplan-Meier statistical analysis of tumor grade (III or IV) in patients with elevated amounts of the miR-301a containing exosomes in the serum showed a shorter overall survival (OS) time in comparison with patients with a lower level (*p* < 0.01). Univariate and multivariate Cox regression analyses verified that the amounts of miR-301a containing exosomes in the serum were individually related to OS. Lastly, they concluded that miR-301a could trigger AKT and FAK signaling through a negative regulatory effect on PTEN. The study showed that serum levels of exosomal miR-301a could indicate variations in glioma patients. The miR-301a containing exosomes in the serum could be an efficient biomarker for GBM diagnosis and prognosis [137].

Manterola et al., [169] isolated exosomes from the sera of 30 patients with GBM and 30 healthy controls. miR-564-3p, miR-320 and RUN6–1 showed the largest variation in expression based on miRNA chip technology, and it was found that RUN6–1 (alone or with other miRNAs) could be used for the diagnosis of GBM. Their study also verified that cancer related miRNAs in serum exosomes could act as biomarkers for the prognosis and monitoring of CNS cancers [170]. Wei et al., [171] utilized a quantitative analysis to determine the exosomal contents of GBM patients for the first time, and found that the exosomes had a relatively abundant content of miRNAs.

Li et al., assessed whether the measurement of miRNAs could monitor the efficacy of radiotherapy in GBM patients [172]. The miRNA contents of serum exosomes were sequenced before and after radiotherapy in a cohort study of GBM patients. The differentially expressed miRNAs, included 18 that were over-expressed and 16 that were down-regulated. Consequently, the target genes of the DE miRNAs were predicted based on various databases. Moreover, it was shown that the target genes were mainly involved in metabolism, the p53 pathway, and tumor progression pathways, which suggested that these miRNAs could play a vital role in the occurrence and progression of glioma, via their effects on target genes. Overall, the study found differences in the exosomal miRNAs present in body fluids in response to radiotherapy, and could be novel biomarkers to monitor the effects of radiotherapy in glioma patients [138, 139, 172].

## Exosomal proteins in glioma

Exosomal proteins have distinctive features compared to other proteins that are employed as biomarkers. For instance, nuclear transcription factor X-box-binding protein 1 (NFX1) and cGMP-dependent protein kinase 1 (PKG1) have only been identified in serum exosomes [173]. Some studies have suggested that H1° histone and EGFRvIII that were transferred by exosomes could accelerate cancer development [174, 175]. On the contrary, another study indicated that PTEN-containing exosomes could inhibit GBM cell progression [176]. Tumor-derived proteins that are freely circulating in blood may be highly diluted, and could be mixed with other similar circulating biomolecules, which could confuse the tests [177, 178]. It was found that many exosomal proteins such as HMGB 1[140], IL-8, PDGFs, caveolin 1, and lysyl oxidase [141], L1CAM [142], STC1, STC2 [143], EGFRvIII [144], VEGF-A [145], CRYAB [146], PTRF [147], PD- 1[148], IL13Rα2, IL13QD [149], **CAV 1**[150], NK-Exo [151], SRSF1, SRSF3 [152], NANOGP8 [153], PTENP1 [155], CLIC1 [156], K-Ras [157], immunoglobulin (Ig) G2 and IgG4 [158], TrkB [159], MGMT mRNA [160], EGFRvIII [161], N-glycoproteins [162], LOX, ADAMTS1, TSP1, VEGF [163], NF-κB [165], Glut-1, HK-2, and PKM-2 [166], TDP-43 [167] were up-regulated. On the other hand some other proteins, such as *IL-8, ZAP70, TGF-β* [148], IFN-gamma, granzyme B [154], and CRCL [164] were down-regulated in glioma tumors (Table 2).

Secondly, exosomal proteins show a greater specificity in comparison with free serum proteins. Glypican-1 (GPC1) which is abundant in the contents of tumor-derived exosomes, showed higher specificity than serum CA-199 or free GPC1 (100% vs. 79.49% vs. 82.14%) to discriminate pancreatic tumor tissue from normal tissue [179]. Thirdly, the encapsulation of proteins inside the membrane of the exosomes make them generally more stable than free proteins, because they are sheltered from degradation by serum proteases and other enzymes [173].

Cell adhesion molecule L1CAM (L1, CD171) stimulates the autocrine/paracrine secretion of various factors, which stimulate the proliferation, migration and invasion of glioma tumor cells. In normal tissue, L1 expression plays a vital role in neuronal development where it is located on the outer membrane of axons, but is also expressed in glioma tumors [24, 180]. L1 possesses an extracellular ectodomain, five fibronectin domains, and 6 immunoglobin-like domains, which are frequently detached and released into the extracellular space. L1 undergoes interactions with different partner proteins, such as L1 itself, integrins, and other outer membrane proteins [181–183]. L1 has a molecular weight of 220 kDa and interacts with integrins through an arginine, glycine, aspartic acid (RGD) domain [184, 185]. Two types of integrins, which stimulate focal adhesion kinase (FAK), and fibroblast growth factor receptor (FGFR) were found to be interacting partners with L1 in glioma tumors [186].

It was reported that L1 (or its ectodomain) that were present in glioma cells could stimulate cell migration [187–190]. Pace et al., showed that small exosomes with L1 on the surface could stimulate glioma cell proliferation, migration, and invasion [142]. Exosomes with L1 on the surface were extracted from the media of the T98G glioma cell line, and their effects on GBM cell lines and primary GBM cells were studied. L1 expressing exosomes increased the migration velocity in 3 cell lines (T98G/shL1, U-118 MG, and primary GBM cells) according to the highly sensitive SuperScratch assay in comparison with L1-low expressing exosomes derived from L1-attenuated T98G/shL1 cells. L1 expressing exosomes also increased proliferation based on cell cycle analysis and cell counting. Furthermore, L1 expressing exosomes caused primary glioma cell invasion in the presence of the non-invasive T98G/shL1 cell line in a chick embryo brain tumor model, but L1-low expressing exosomes did not. The migration and cell proliferation stimulated by L1, was reduced by inhibitors of focal adhesion kinase (FAK) and FGFR, to different degrees. Both L1 expressing exosomes as well as the soluble ectodomain of L1, could stimulate migration, proliferation and invasion in glioma cells [142].

The SASH1 gene is commonly expressed in normal tissue. The SASH1 protein is involved in cell growth, proliferation, and apoptosis, and has been shown to play a role in the progress of different diseases. SASH1 is considered to be a tumor suppressor gene, because it is absent or shows reduced function in many cancer types, such as lung cancer [191], gastric cancer [192], colon cancer [193, 194], cervical cancer [195], ovarian carcinoma [196], and thyroid cancer [197]. Previously, it was found that SASH1 showed lower expression levels in high-grade glioma tissue samples in comparison with low-grade samples Reduced expression of SASH1 has been correlated to poor prognosis [198]. Conversely, up-regulated expression of SASH1 in GBM was correlated with lower levels of proliferation and invasion [199].

Wu et al., reported that SASH1 gene knock-down in cultured astrocytes considerably reduced cellular adhesion and increased invasion [200]. Likewise SASH1 up-regulation in the C6 cell line increased cell adhesion and reduced invasion. Moreover, expression of the integrin β8 was considerably lower in SASH1-down-regulated astrocytes, and was increased in SASH1 over-expressing C6 cells. In addition, DNA methylation and ChIP assays indicated that the SASH1 gene was more methylated in the C6 cell line than in astrocytes. Moreover, HMGB1 was able to interact with the CpG islands in the SASH1 gene. Up-regulation of HMGB1 in astrocytes caused hyper-methylation of the SASH1 gene. This study showed that HMGB1 was involved in SASH1 gene methylation, and that methylation reduced the expression of the SASH1 and integrin β8 genes, resulting in decreased cell adhesion and increased cell migration [200].

Ma et al., studied various protein expression patterns of normal glial cell and glioma-derived exosomes, and the effects of SASH1 gene expression in glioma [201]. They isolated exosomes from astrocytes and C6 cells, and identified their exosomal proteins using mass spectrometry. The results of gene ontology (GO) and Kyoto encyclopedia of genes and genomes (KEGG) analysis showed that there were various groups of unique proteins in exosomes from normal glial cells and glioma cells. In normal cells, the chief clusters were mostly involved with RNA transcription and proteins, whereas in glioma cells the top clusters were involved in activation of the PI3K-Akt pathway, adhesion, and tumorigenesis pathways. Western blotting indicated that although HMGB1 was present at a low level in exosomes secreted from cultured astrocytes, it was significantly up-regulated in the C6 cell line. Moreover, astrocyte-derived exosomes could increase the expression of SASH1 in C6 cells, although the exosomes secreted from HMGB1-low astrocytes could not. Recombinant HMGB1 caused down-regulation of SASH1, while TLR4 expression was enhanced. HMGB1 is an extracellular protein that normally down-regulates SASH1, but when it is contained in exosomes it up-regulates SASH1. However, the aforementioned process, which was suggested to be related to TLR4 signaling, needs more research. The structure-dependent function of the secreted protein HMGB1 to stimulate or suppress tumorigenesis, opens a new horizon for understanding the interaction between tumor cells and their microenvironment [201].

Hypoxia is an important factor that can disturb the integrity of the blood-brain barrier (BBB). The BBB is composed of specific brain microvascular endothelial cells (BMVECs) that are joined together by tight junction complexes. The BBB acts to protect the microenvironment of the central nervous system (CNS) from external toxins or infectious pathogens. However, hypoxia disrupts the tight junctions of the BBB. The disrupted tight junctions between the BMVECs in GBM patients, results in the pathological opening and outflow from the BBB [202–204]. However, the mechanism of BBB disruption in GBM patients has not been fully elucidated. Some studies have shown that exosomes secreted from GBM cells contain various pro-angiogenic factors that are needed for proliferation and migration of endothelial cells [205]. Vascular endothelial growth factor (VEGF) is considered to be the best-known pro-angiogenic factor, and was found to be present in GBM secreted exosomes, although the role of exosomal VEGF in the BBB opening is not fully understood [205, 206].

Zhao et al., showed that GBM-secreted exosomes could induce the disruption of the BBB in laboratory studies [145]. They found that the expression of VEGF-A was up-regulated in GBM secreted exosomes in hypoxic conditions, which increased the leakiness of an in vitro BBB model via suppressing the expression of claudin-5 and occluding proteins. An in vivo leakiness assay indicated that the secreted exosomes from hypoxic GBM tumors remained active during circulation, and caused leakiness to develop in the BBB [145]. In CNS, the protein known as cryAB/HspB5 (αB-crystallin or small heat shock protein B5) is normally expressed in astrocytes and oligodendrocytes [207]. In GBM, cryAB is over-expressed in the brain [208, 209] and inhibits apoptosis through binding and suppressing caspase-3 [208–210]. cryAB expression is up-regulated in various neurodegenerative diseases including Parkinson’s disease, Alzheimer’s disease, multiple sclerosis, amyotrophic lateral sclerosis, age-related macular degeneration and traumatic brain injury, and is found to accumulate in astrocytes and oligodendrocytes of the CNS [211, 212]. cryAB has also been found in the extracellular matrix adjoining retinal cells [213, 214], and it was also found that presentation of cryAB epitopes could lead to stimulation of T-cells [215, 216]. Both of these observations suggested that cryAB was an important intracellular protein in the CNS. It was then found that cryAB was also a secreted protein, and it was further suggested that exosomes could mediate its secretion [146, 213, 217–219].

Phosphorylation of cryAB mediated an interchange between the monomeric and oligomeric states of the protein, and affected its function [220–223]. Related to secretion of cryAB by exosomes, a bioinformatics analysis on its sequence using SecretomeP 2.0 [224] and SignalP 4.1 [225] indicated that a signal peptide(s) were required. Kore et al., found that the majority of the cryAB molecules in exosomes were non-phosphorylated [226]. Large cytosolic inclusion bodies were created after transfection of cryAB-free glioma cells with a yellow fluorescent protein (YFP)-tagged triple phosphomimic (3-SD) cryAB construct. This study showed that phosphorylation considerably decreased the secretion of cryAB in exosomes. Moreover, they found that inhibition of the O-GlcNAcylation of cryAB also reduced its co-localization with CD63 and Rab27, resulting in the decreased secretion of exosomes. Hence, it was suggested that O-GlcNAcylation and lack of phosphorylation were both involved in the loading and secretion of cryAB in exosomes [226].

## Conclusions

Exosomes play a vital role in glioma tumor biology, immunology, and chemo-sensitivity and can act as biomarkers for glioma diagnosis. The miRNAs and proteins contained in exosomes have played a critical role in diverse cancers, including glioma. The packing of miRNAs into exosomes is a selective process. The levels of individual miRNAs and proteins inside exosomes are altered during tumorigenesis. Based on these data, the miRNA and protein contents of exosomes could be used as a new type of biomarker for the diagnosis and monitoring of treatment response of gliomas. Furthermore, the diagnostic efficiency of the miRNA and protein contents of exosomes could be superior to more commonly employed biomarkers. Exosomes can transfer miRNAs and proteins between tumor cells for the transmission of information and to mediate signaling pathways. miRNA and protein-containing exosomes can modulate tumor progression and metastasis, and could likewise play a critical role in the immune responsiveness and chemo-sensitivity of tumors. Aberrant expression of exosomal miRNAs and proteins has been reported in several cancers, including gliomas, and is implicated in glioma pathogenesis and progression, suggesting their possible application in diagnosis, prognosis and therapy. In this review, the role of several exosomal miRNAs and proteins that regulate various oncogenes and tumor suppressor genes involved in glioma development, their prospective roles as prognostic and diagnostic markers and their therapeutic targets were summarized.

Furthermore, exosomes could be potentially used to transfer chemotherapeutic drugs and biotherapeutic agents to various cells and tissues. Thus, engineered exosomes could play a future role as efficient delivery vehicles for direct targeting of glioma tumor cells. Nowadays, TMZ and cisplatin are often used for chemotherapy of glioma, but a method to overcome the development of chemo-resistance to these drugs is required, which may involve intervention to modulate miRNAs and exosomes. More studies are required to fully identify the specific miRNA and protein contents of exosomes, and their mechanism of action in various cancers, including gliomas.

## Abbreviations

3-SD: Triple phosphomimic
A: Adenine
AGAP2: Ankyrin repeat and PH domain 2
AGAP2: Arf GTPase-activating protein-2
Ago: Argonaute
Arf GAPs: ADP-ribosylation factors GTPase-activating proteins
BBB: Blood-brain barrier
BMVECs: Brain microvascular endothelial cells
C: Cytosine
CA-19-9: Cancer antigen 19–9
CADM1: Cell adhesion molecule 1
cAMP: Cyclic adenosine monophosphate
CAV1: Caveolin-1
cGMP: Cyclic guanosine monophosphate
CNS: Central nervous system
Cox: Cyclooxygenase-1
cryAB: Alpha-crystallin B
cryAB/HspB5: HSPB5 (also known as CRYAB or αB- crystallin) is a small molecular weight heat shock protein (sHSP)
CSF: Cerebrospinal fluid
CYP2J2: Cytochrome P450 2 J2
ESCRT: Endosomal sorting complexes required for transport
EVs: Extracellular vesicles
exo-miR-301a: Exosomal miR-301a
Exo: Exosomal
EZH2: Enhancer region of Zeste 2
FAK: Focal adhesion kinase
FGFR: Fibroblast growth factor receptor
G: Guanine
GBM: Glioblastoma multiforme
GBM: Glioblastoma
GEXs: Glioma-derived exosomes
GLUT: Glucose transporter
GO: Gene ontology
GPC1: Glypican-1
GTP: Guanosine-5′-triphosphate
H-GEXs: Hypoxia-induced GEXs
Hbp1: High-mobility group box transcription factor 1
HER2: Human epidermal growth factor receptor 2;HMGB1 high mobility group box 1 protein
hMSCs: Human mesenchymal stem cells
Hsp: Heat shock proteins
IDH^MUT^: Isocitrate dehydrogenase mutant
IL: Interleukin
ILVs: Intraluminal vesicles
K +: Potassium
KEGG: Kyoto encyclopedia of genes and genomes
L1CAM: L1 cell adhesion molecule
MDSCs: Myeloid-derived suppressor cells
miRNA: MicroRNAs
MSCs: Mesenchymal stem cells
mTOR: Mammalian target of rapamycin
MVB: Multivesicular bodies
MVE: Multivesicular endosome
N-GEXs: Normoxia-induced GEXs
NFX1: X-box-binding protein 1
NHEJ: Non-Homologous End Joining
NO: Nitric oxide
OS: Overall survival
PDCD4: Programmed cell death 4
PKG1: cGMP-dependent protein kinase 1
pri-miRNAs: Primary miRNAs
Prkar1a: Protein kinase cAMP-dependent type I regulatory subunit alpha
PTEN: Phosphatase and tensin homolog
qPCR: Quantitative polymerase chain reaction
RBP: RNA-binding proteins
RECK: RGD: Arginine, Guanine and Aspartic acid
RNA-ChIP: RNA chromatin immunoprecipitation
RNase: Ribonuclease
ROS: Reactive oxygen species
SASH1: SAM and SH3 domain-containing 1
shRNA: Short hairpin RNA
sncRNAs: Small non-coding RNAs
snoRNA: Small nucleolar RNAs
STAT3: Signal transducer and activator of transcription 3
T: Thymine
TCEAL7: Transcription Elongation Factor A Like 7
TCGA: The Cancer Genome Atlas
TGF-β: transforming growth factor β
TGN: Trans-Golgi Network
TIMP3: Tissue inhibitor of metalloproteinases-3
TLR4: Transmembrane lipopolysaccharide receptor
TMZ: Temozolomide
tRNA: Transfer RNA
U: Uracil
VEGF: Vascular endothelial growth factor
Wnt: Wingless-related integration site
XRCC4: X-ray repair cross-complementing
YFP: Yellow fluorescent protein
